# High-pass-filter cut-offs optimization of the filter-based fatigue index during dynamic contractions

**DOI:** 10.1186/1757-1146-7-S1-A129

**Published:** 2014-04-08

**Authors:** Jungyoon Kim, Sunwoo Park, Youngho Kim

**Affiliations:** 1 Department of Biomedical Engineering and Institute of Medical Engineering, Yonsei University, Wonju, Gangwon, 220-710, Korea

## Background

Traditional muscle fatigue indices (F_mean_ and F_med_) have a relatively low sensitivity under dynamic exercise conditions. Various muscle fatigue indices have been attempted to overcome this problem [1,2]. However, their methods required large amounts of computation and had limitations in time-frequency resolution [3]. Kim et al. introduced the use of a filter-based fatigue index (FI_hlrOPT_), which the ratio of high-frequency to low-frequency components of EMG power [3]. In this study, we optimized the cut-offs of the high-pass-filter (HPF) to maximize the correlation coefficient between the peak power and the FI_hlrOPT_ in different muscles, and then to determine the frequency bandwidth of our fatigue index.

## Methods

Forty-one healthy males were recruited for this study. Twenty-seven subjects performed knee extension/flexion exercises and fourteen subjects performed elbow flexion/extension exercises on an isokinetic dynamometer (Biodex System 3, Biodex Medical Systems, NY, USA). 10 repetition maximum was used for fatigue exercises. The experimental protocol was 5 sets of 10 knee extensions and elbow flexion with 2 minutes of rest between sets. EMG signals were obtained from rectus femoris (RF), vastus medialis (VM), vastus lateralis (VL) and biceps brachii (BB) muscles using the Noraxon EMG System (MyoSystem 1200, Noraxon Inc., AZ, USA). EMG signals, as well as biomechanical signals (angle, angular velocity and torque) were simultaneously recorded with a sampling rate of 1kHz. FI_hlrOPT_ was calculated and cut-offs of HPF were optimized to maximize the correlation between the peak power and FI_hlrOPT_.

## Results

Optimized cut-offs of RF, VM, VL and BB were similar (353.3± 49.5 Hz, 343.9± 34.2 Hz, 353.7± 36.1 Hz and 362.3± 28.2 Hz). RF, VM, VL and BB muscles showed good correlation with joint power (correlation coefficient was 0.81± 0.08, 0.56± 0.23, 0.52± 0.24 and 0.72± 0.08).

## Discussion

Similar to previous study, cut-offs of HPF of muscles were about 350Hz (RF: 360Hz in previous study). Similar cut-offs of HPF in different muscles showed the possibility of general cut-off for muscle fatigue estimation. Mills showed that the compound muscle action potential spectrum did not change during fatigue above 200Hz [4]. Thus, the high frequency power decreased during fatigue because of the reduced motor unit activation. The low frequency power increased during fatigue because of the elevated motor unit action potential areas. FI_hlrOPT_ could reflect the decrease in the peak power during fatigue by these reasons.

**Figure 1:**
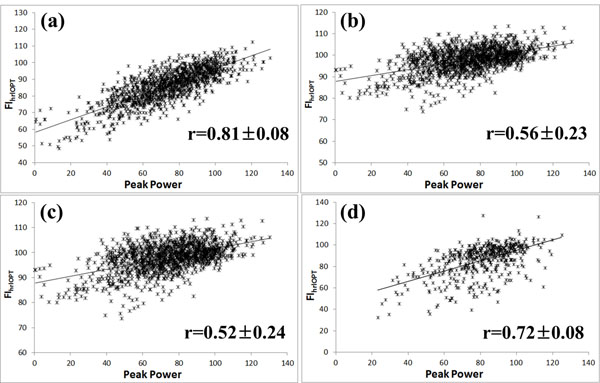
Linear representations of peak power output versus various EMG indices for all subjects. Peak power output versus FI_hlrOPT_ of (a) RF muscle; (b) VM muscle ; (c) VL muscle ; (d) BB muscle
